# Improved artificial hummingbird algorithm for electric vehicle charging station fast and slow charging location determination method

**DOI:** 10.1371/journal.pone.0332872

**Published:** 2025-09-30

**Authors:** Sixia Fan, Xiangyu Zeng, Lanxin Li, Shuqi Xu

**Affiliations:** School of Business, Shanghai Dianji University, Shanghai, China; Vellore Institute of Technology, INDIA

## Abstract

Electric vehicle (EV) charging infrastructure is rapidly improving. To address high site selection costs from unbalanced fast-slow charging ratios and multi-party cost allocation issues, we propose a four-objective optimization model based on a three-party cost game (suppliers, users, power grid). The model minimizes: (1) construction/operation costs, (2) user time loss costs, (3) grid power loss costs, and (4) voltage deviation costs under different charging modes. An improved artificial hummingbird algorithm solves the model. Results show the approach improves economic efficiency, provides valuable reference for EV charging station siting, and demonstrates strong algorithm robustness and generalization.

## 1. Introduction

As global energy demand continues to rise, particularly the reliance on non-renewable energy sources like coal and oil, environmental pollution has worsened, damaging the atmosphere and posing severe challenges to living conditions. Consequently, the concept of “carbon neutrality” [1] has emerged. The green and low-carbon transition of China’s energy system serves as a fundamental pathway to realize the nation’s Dual Carbon objectives – reaching carbon emission peak before 2030 and attaining carbon neutrality by 2060 [2]. By the end of 2023, China’s pure electric vehicle fleet had reached 15.52 million units, yet the construction of charging infrastructure still lags behind the “1:1 vehicle-to-charger ratio” target [3] set by the National Energy Administration, becoming a bottleneck for industry development. As a pivotal driver of the “dual carbon” strategy, electric vehicles have demonstrated significant emission reduction effects. With the increasing production and sales of electric vehicles, China has introduced a series of policy measures to promote the popularization and development of EV charging stations. The “Development Plan for Electric Vehicle Charging Infrastructure (2015-2020)”, issued in 2015, explicitly called for accelerating charging infrastructure construction to meet demand. This initiative was later incorporated into the 2020 Government Work Report to boost the growth of new infrastructure industries [4] On January 10, 2022, the National Development and Reform Commission and other departments issued the “ Implementation Opinions on Further Enhancing the Service Support Capacity of Electric Vehicle Charging Infrastructure ”, which significantly boosted the development of the new energy vehicle charging market. This policy established charging as the mainstream solution while positioning battery swapping as a supplementary approach [5–6].

Therefore, scientifically and rationally planning the siting and capacity allocation of charging stations not only guarantees a convenient and efficient charging experience for users, preventing queuing caused by insufficient facilities, but also comprehensively improves service quality and user satisfaction, thereby significantly increasing public acceptance of electric vehicles. This issue has already attracted systematic attention and in-depth research from numerous scholars both at home and abroad.

## 2. Literature review

With the rapid development and widespread adoption of electric-vehicle technology, the siting of charging stations has become a focal concern for both academia and industry. Research on this issue not only determines the charging convenience of EV users, but also directly affects the operational efficiency of charging stations, the stability of the power grid, and environmental sustainability.

Early studies, such as Hamed et al. [7], adopted a maximum-coverage location model to address EV charging-station siting under expected daytime and nighttime demands, integrating financial feasibility to improve adoption rates. As attention shifted toward user behavior and psychological factors, Xu et al. [8] incorporated range anxiety and route-choice issues caused by battery degradation, aiming to minimize drivers’ perceived range anxiety. Wu and Sioshansi [9] innovatively proposed a classical stochastic-flow-capturing location framework that optimizes station placement as the number of charging stations increases. Jung et al. [10] introduced a dynamic siting model that replaces flow interception with trip interception, effectively reducing user queueing delays and service-request rejection rates and thereby enhancing station service levels. More recently, the rapid advancement of big-data analytics and artificial-intelligence techniques has opened new avenues for EV charging-station siting. Tu et al. [11] note that charging-station locations are constrained by dynamic travel patterns and charging durations; to tackle this, they extract massive GPS trajectory data to build a multi-objective siting model that maximizes total travel distance covered and minimizes charging-queue waiting time. Keawthong et al. [12] analyze Bangkok EV GPS data and propose a data-driven process that minimizes the aggregate travel time from EVs to charging stations. Several studies further adopt a stakeholder-oriented perspective: Ko et al. [13] argue that station locations must simultaneously satisfy users, government, and charging-service providers, and thus put forward a trajectory-based siting model balancing these interests. Frade et al. [14] take Lisbon as a case study and construct a maximum-coverage charging-station model by integrating local population and employment density characteristics. Recognizing the limited driving range of EVs and the uncertainty of their initial state of charge, He et al. [15] develop a bi-level charging-station siting model that explicitly accounts for range limitations and stochastic initial SOC to optimize deployment strategies. Shahraki et al. [16] adopt total vehicle-kilometers-traveled as the objective and find that, as the total number of charging stations increases, the optimal locations expand from the inner city outward, exhibiting diminishing marginal benefits. Against the backdrop of sustainable development and environmental protection, Hosseini & Sarder [17] develop a Bayesian-network model that integrates both quantitative and qualitative factors; from this sustainability perspective they formulate a suite of sub-indicators to provide a novel view on charging-station siting. Namdeo et al. [18] propose a geospatial modeling approach that derives a siting plan based on users’ travel patterns and EV usage intensity.Additional studies have explicitly considered the interplay between the power grid and the traffic network. Hidalgo et al. [19] compute spatiotemporal energy demands while accounting for individual user requirements, constructing a dedicated siting model that minimizes (i) the total infrastructure installation cost and (ii) the volume of failed trips due to insufficient on-board energy; they further analyze how variations in user charging behavior and battery energy efficiency affect algorithmic outcomes. Tadayon et al. [20] present a design strategy that simultaneously considers the electric grid and the urban transportation network; their multi-objective charging-station siting model maximizes user travel comfort, minimizes all station-related cost indicators, and satisfies constraints such as traffic volume and charging duration. Jing Xiaomin [21] systematically incorporates traffic-network layout, distribution-system efficiency, and multiple cost dimensions into a comprehensive multi-objective planning model for charging-station siting.

Research on the siting of EV charging stations has achieved substantial advancements. Early investigations predominantly employed coverage-based and flow-capture models, whereas recent efforts have increasingly leveraged large-scale data and artificial-intelligence techniques for optimization. Contemporary studies further integrate stakeholder perspectives, sustainability criteria, and coordinated analyses of power-grid and transportation networks. Nonetheless, the extant literature remains largely focused on station-level cost constraints—comprising construction and operation-and-maintenance expenditures—and subsequently derives user routing patterns and range-anxiety considerations, ultimately yielding single- or two-party benefit maximization. In capacity optimization, existing approaches typically address the charging station as an aggregate entity, neglecting flexible allocation between fast and slow chargers. Concurrently, investigations incorporating the operational implications of charging-station integration into distribution networks remain scarce, despite the significant stresses imposed by high-power fast-charging technologies. Consequently, a multi-cost, multi-dimensional, and multi-objective framework for siting and sizing represents the prevailing trajectory for future EV charging-station planning and decision-making.

In recent years, capacity sizing has been explicitly incorporated into optimal siting studies for new-energy charging infrastructure. Liu and Lin [22], grounded in the P-median principle, developed a combined location-and-capacity model that simultaneously minimizes user detour costs, fixed investment expenditures, and operation-and-maintenance outlays for EV charging stations. Peng et al. [23] constructed a comprehensive charging-station cost framework that integrates land-use fees, equipment procurement costs, and long-term O&M expenses, and they employed an efficient search strategy to explore the solution space. A numerical case study under their framework yielded optimal siting and capacity configurations under given constraints.To expedite optimization and mitigate computational complexity, researchers have increasingly adopted heuristic algorithms for such problems [24]. These algorithms, formulated as near-optimal rather than strictly optimal approaches, can rapidly approximate globally satisfactory solutions. Commonly used techniques include genetic algorithms and particle swarm optimization (PSO). Wang Huanlin [25] formulated a charging-demand allocation model based on candidate station locations and solved it via PSO to identify sites that satisfy traffic convenience, operational economy, regional development prospects, grid security, and construction feasibility. Wang Yong [26] established a cost-minimization siting-and-sizing model and applied an improved mutation-based PSO to obtain solutions. Qu Rui et al. [27] introduced a chaotic simulated-annealing PSO that exploits probabilistic jumps to escape local optima and locate global solutions within the search space.Despite these algorithmic enhancements, PSO remains susceptible to premature convergence, which can degrade solution quality in location-and-capacity models. Addressing this limitation remains a critical challenge for heuristic approaches to EV charging-station planning.

In conclusion, this paper presents a multi-dimensional, multi-objective model for simultaneously siting electric-vehicle charging stations and determining their capacity. By explicitly balancing the cost concerns of suppliers, users, and grid operators, the model formulates four objective functions: (i) minimization of charging-station construction cost, (ii) minimization of users’ annual charging-related travel cost, (iii) minimization of grid power losses, and (iv) minimization of voltage deviation. This integrated framework remedies the incompleteness of conventional siting objectives, enhances the flexibility of fast- versus slow-charger allocation, and fills the gap in existing literature regarding the heterogeneous impacts of mixed charger types on power-system performance. To mitigate the issues of excessive iterations and the elusiveness of global optima, this paper proposes a chaotic non-uniform mutation-enhanced artificial hummingbird algorithm (CAHA) specifically tailored for solving the multi-dimensional, multi-objective siting model. The algorithm enhances the conventional Artificial Hummingbird Algorithm via three optimization strategies while integrating chaos theory to improve global exploration capability. A case study in the New-City Planning Area of Xi’an demonstrates that CAHA surpasses AHA, PSO, and NSGA-II in both total cost reduction and solution robustness, thus providing a practical and cost-effective blueprint for supporting China’s “Dual-Carbon” 2030/2060 targets.

## 3. Establishment of a multi-objective location model for electric vehicle charging stations

**Fig 1:**
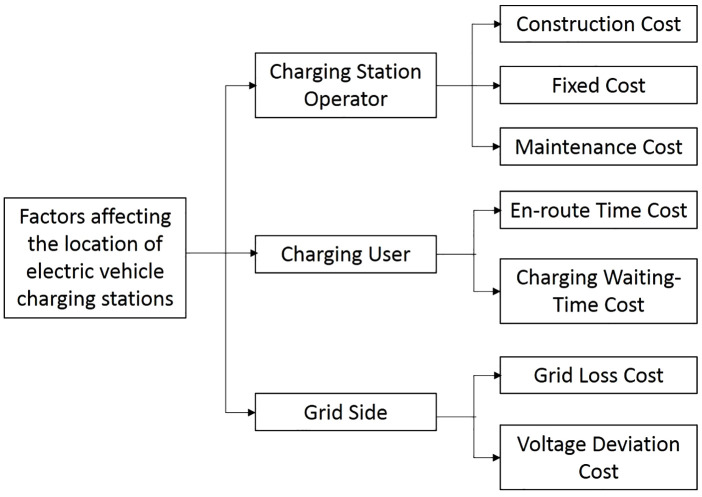
Factors affecting the location of electric vehicle charging stations.

In the real world, the siting of electric-vehicle (EV) charging stations is influenced and constrained by a multitude of factors, including national policies, geographical conditions, and the spatial distribution of actual EV user charging demands. This study primarily examines the perspectives of the operator, end-user, and grid side. By examining the multidirectional gaming among these three parties, it pursues the minimization of operator costs and the maximization of user satisfaction while simultaneously ensuring the minimization of grid loss costs. From the operator perspective, cost factors primarily comprise expenditures on station deployment, land acquisition, road construction, labor, procurement of charging equipment (e.g., charging piles), and subsequent operational and maintenance costs of charging infrastructure. Operators seek to fulfill EV charging demands at minimal feasible expenditure. From the end-user perspective, analysis focuses on annual loss-induced costs during charging processes. This encompasses: (1) temporal costs incurred en route to charging stations—user experience improves proportionally with proximity between origin points and charging facilities; (2) queuing-induced temporal costs during charging sessions. Higher station density within a region, greater aggregate charging pile availability, and elevated proportions of fast-charging equipment collectively enhance demand fulfillment capacity, thereby reducing queue times and diminishing users’ charging wait-time costs. As the demand for fast-charging stations for EVs continues to surge, the burden on the grid side is also increasing. Therefore, by optimizing line losses and charging losses, the grid-side network loss costs can be effectively reduced. Fig 1 illustrates the key factors influencing the siting of EV charging stations.

### 3.1. Factors influencing the location selection of electric vehicle charging stations

#### 3.1.1. Operator Side: Charging station construction costs.

Charging station siting is influenced by multiple factors, among which construction cost [28] is one of the most critical considerations. a) Land cost is the foremost element, serving as a key component of overall construction expenses; therefore, it must be fully integrated into the siting decision. b) Infrastructure development costs including power-supply facilities and road connections—are also pivotal, as geographic location directly affects the investment required for these assets. c) Construction and labor costs further shape site selection; regions with relatively low labor costs can significantly reduce both initial outlay and ongoing O&M expenses. d) Rights and permitting fees must not be overlooked, since certain jurisdictions impose specific charges for land-use rights, building permits, and other regulatory approvals. Finally, e) market demand and profit expectations are decisive. Even if construction costs are low, a site lacking charging demand or facing intense competition may fail to deliver projected returns.

Hence, a comprehensive evaluation of these factors is essential to ensure that charging stations are built in the most cost-effective and strategically advantageous locations.

#### 3.1.2 User Side: En-route loss cost incurred by users.

The en-route loss cost for users encompasses both travel-time cost [29] and queuing-time cost, both of which translate into losses of users’ time and resources.

a) Travel-time cost captures the economic value of the time required for an EV to reach a charging station. It integrates multiple factors: the distance between the charging station and the demand origin, the urban travel-time cost coefficient, road-condition complexity that limits vehicle speed, the number of operating days per year, and the probability that a given vehicle will choose that station. By synthesizing these elements, the monetary value of travel time during the charging journey can be quantified, providing an essential input for cost–benefit analyses and operational decisions. b) Queuing-time cost refers to the average waiting time at the station before charging service begins. It is calculated from the station’s workload—defined by the EV arrival rate relative to the charging rate—and the station’s capacity (i.e., the number of chargers). Estimating the expected waiting time allows an assessment of station efficiency and supports adjustments in charger deployment to balance service quality against investment cost.

#### 3.1.3. Grid Side: Impacts on the power system.

Laboratory simulations demonstrate that different EV charging/discharging patterns distinctly alter the daily load profile of the grid:

a) Uncontrolled charging amplifies peak-hour demand, intensifying the challenge of peak-shaving.b) Controlled charging produces a milder impact, mitigating—though not eliminating—the peak-shaving pressure created by uncontrolled patterns.c) Controlled charging-and-discharging (V2G) actively flattens the load curve, achieving genuine peak-shaving and valley-filling.

As EV penetration rises, the grid’s characteristics evolve: the minimum daily load increases, the maximum load decreases, and the peak-valley difference narrows. These shifts confirm that all charging modes materially affect the grid and complicate peak regulation.Consequently, during charging-station siting, priority must be given to solutions that minimize grid losses, thereby enhancing the economic efficiency of power-system operation.

### 3.2. Siting model formulation

#### 3.2.1. Problem assumptions.

To facilitate the construction of an electric-vehicle charging-station siting model, the following assumptions are made, as summarized in Table 1.

**Table 1:** Problem hypothesis.

Category	Assumptions
Demand	(1) The charging distance between a charging station and a demand node is treated as the straight-line (Euclidean) distance between the two points. (2) EV users are assumed to prefer the nearest charging station. (3) Every user is assumed to require the same amount of electricity for each charging session.
Charging Station	(1) All fast chargers and slow chargers within any given station are assumed to have identical electrical parameters. (2) Each charger can serve only one electric vehicle at a time.
Electric Vehicle	(1) All vehicles are assumed to have identical charging efficiency when operating under the same electrical specifications. (2) Every electric vehicle originating from the same demand node is assumed to charge at one and the same charging station.

#### 3.2.2. Objective function.

To simultaneously reduce (i) users’ en-route loss costs, (ii) operators’ construction-and-operation costs, and (iii) grid-side network-loss costs, this paper formulates a multi-objective mathematical model.

(1) Operater side: Charging station construction costs

The cost of charging station operators can be divided into construction cost $C_{\text{con}}$ and equipment maintenance cost $C_{\text{op}}$, Based on the different prices of fast and slow charging, the following mathematical formula is established:


$$C_{\text{con}}=\frac{{r_{\text{0}}\left(\text{1}+r_{\text{0}}\right)}^{n_{year}}}{{\left(\text{1}+r_{\text{0}}\right)}^{n_{year}}-\text{1}}\left(C_{g}+\varphi n_{ch}^{\text{2}}+(\varepsilon_{\text{1}}(\text{1}-b)+\varepsilon_{\text{2}}(b))n_{ch}\right)$$ (1)

In the formula, $C_{\text{con}}$ is the construction cost; $r_{O}$ is the discount rate, which is 0.08; $\;n_{year}$ is the operating life, which is 20 years; $\;C_{\text{g}}$ is the fixed investment cost, which is 3 million yuan; $\;n_{ch}$ is the number of charging piles in the charging station; ϕis the equivalent investment coefficient of the distribution transformer and transmission line related equipment cost, which is 20,000 yuan/unit;  ϵ is the unit price of the charging pile, which is 200,000 yuan/unit for fast charging and 100,000 yuan/unit for slow charging.


$$C_{\text{op}}=\left(C_{g}+\varphi n_{ch}^{\text{2}}+(\varepsilon_{\text{1}}(\text{1}-b)+\varepsilon_{\text{2}}(b))n_{ch}\right)\gamma$$ (2)

$C_{\text{op}}$ is the equipment maintenance cost; γ is the conversion coefficient of labor and equipment operation and maintenance cost, which is taken as 0.1.

In summary, the objective function of the construction and operation cost of the charging station is:


$$f_{\text{1}}=\sum_{i=\text{1}}^{I}\left(C_{\text{coni}}+C_{\text{opi}}\right)$$ (3)

*I* is a collection of charging stations.

(2) User side: The annual loss cost of users during charging journey

In formulating the objective function for this study, queueing theory [30–31] is employed as a fundamental analytical tool. Queueing theory studies the performance and behaviour of queueing systems, in which customers arriving at a service facility must wait until they are served. Its purpose is to build mathematical models that evaluate system performance indicators—such as average waiting time, average service time, and system utilisation—and to furnish guidelines for optimising system design and operation. The objective function developed herein is closely aligned with queueing-theoretic principles. Specifically, it decomposes user-side losses into two queueing-related components: (i) travel time to the charging station, and (ii) waiting time at the station. By embedding these components as sub-objectives within the overall optimisation framework, the model yields a queueing-theory-based function that minimises users’ annual aggregate loss associated with the entire charging journey.

a) Travel-time cost en-route is expressed as:

$$C_{VT}=\text{3}\text{6}\text{5}\beta \frac{\sum_{i=\text{1}}^{I}\sum_{j=\text{1}}^{N_{ch}}pn_{j}\lambda_{ij}d_{ij}}{\nu }$$ (4)

$C_{VT}$ is the time cost of charging; β is the time cost coefficient of urban travel, which is 17 yuan/h; $d_{ij}$ s the Euclidean distance from demand point  j to charging station  i; ν is the average speed of urban traffic, which is 20 km/h;  p is the charging probability of electric vehicles, which is 0.05.

b) Charging waiting-time cost

This paper focuses on siting charging stations that provide both fast and slow chargers. The resulting user waiting experience differs markedly from that of single-mode stations, so the distinct characteristics of fast and slow charging must be explicitly modeled. When a station is equipped with both fast and slow chargers, the waiting times experienced by users will vary according to the mix of charger types. Introduced the concept of equivalent coefficient for fast and slow charging. Among them, the charging service intensity can be expressed as:


$$r_{o}=\frac{{(K}_{m}b+\text{1})\lambda }{m_{\mu }N_{ch}}$$ (5)

among them, $K_{m}$ is the equivalent coefficient of fast and slow charging.

The fast-to-slow charging coefficient is an indicator used to compare how the charging rates of different chargers affect battery charging efficiency. It is defined as the ratio of the effective amount of charge delivered to the battery by a fast charger versus a slow charger within the same time period. In this paper, an equivalent coefficient that combines both charger types is employed. A weighted average is applied to the fast- and slow-charging efficiency coefficients, using their respective usage frequencies or importance as weights, yielding a comprehensive efficiency coefficient that represents the combined charging efficiency of fast and slow chargers.

EV charging stations are equipped with two types of charging stations: fast and slow. The former charges quickly but at a higher cost, while the latter charges slowly and at a lower cost. Therefore, when designing a charging station, it is necessary to consider the ratio of fast charging and slow charging to balance charging efficiency and cost.


$$C_{WT}=\text{3}\text{6}\text{5}\beta \sum_{i=\text{1}}^{I}W_{qi}\sum_{j=\text{1}}^{N_{ch}}pn_{j}$$ (6)

$$W_{q}=\frac{N_{ch}\rho^{N_{ch}+\text{1}}P_{z}}{\lambda N_{ch}!{\left(N_{ch}-\rho \right)}^{\text{2}}}$$ (7)

$$\begin{array}{c} P_{z}={\left[\sum_{k=\text{0}}^{N_{ch}-\text{1}}\frac{\rho^{k}}{k!}+\frac{N_{ch}\rho^{N_{ch}}}{\lambda N_{ch}!\left(N_{ch}-\rho \right)}\right]}^{-\text{1}} \end{array}$$ (8)

Wherein, $C_{WT}$ is the waiting time cost for charging, $\;W_{q}$ is the expected waiting time for electric vehicles in queue, $N_{ch}$ is the number of charging piles at a certain station; $\rho =\frac{\lambda }{\mu }/N_{ch}$ is the number of electric vehicles arriving at the charging station per unit time; p is the charging probability of electric vehicles, which is 0.05; $\;t_{c}$ is the charging time period for electric vehicles, which is 4h; $\mu =\text{1}/t_{s}$ is the average service rate of the charging piles; $\;t_{s}$ is the service time of the charging piles, which is 12h; $P_{z}$ is the probability that all charging piles are idle.

In summary, the objective function of the user’s loss cost during the charging journey each year is:


$$f_{\text{2}}=\sum_{i=\text{1}}^{I}\left(C_{\text{VTi}}+C_{\text{WTi}}\right)$$ (9)

(3) Grid side: network loss costs and voltage deviation costs

In the power system, components such as transmission lines and transformers experience energy loss in the form of thermal energy due to the presence of resistance and reactance when current flows through them. This loss not only wastes electrical energy resources, but also affects the overall efficiency of the system. Reducing network losses has become one of the key objectives for optimizing the operation of the power system. When selecting the location for electric vehicle charging stations, it is necessary to connect the charging loads of each charging station to the distribution system, calculate the active power loss through power flow, and combine the charging electricity price and the operation time of the charging station to ultimately obtain the cost of the objective function three network losses [32]:


$$f_{\text{3}}=\text{365}t_{CH}C_{fa}P_{loss}$$ (10)

$t_{CH}$ is the daily operation time of the charging station, which is 12 hours/day; $\;C_{fa}$ the electricity price, which is 0.8 yuan/kWh; $\;P_{loss}$ is the active power loss.

In the power system, voltage offset refers to the difference between the voltage at a node and the rated value. Excessive or insufficient voltage offset can cause equipment damage or a decrease in power quality, so it is necessary to maintain the voltage within an appropriate range. Minimizing the cost of voltage offset [33] means minimizing the voltage offset at the nodes as much as possible, so that the system operates at the optimal voltage state.

In the site selection of electric vehicle charging stations, it is necessary to obtain the equivalent voltage offset price, and then obtain the objective function of minimizing the cost of four voltage offsets:


$$f_{\text{4}}=\text{365}t_{CH}C_{\mu }\sum_{i=\text{1}}^{N}\left|U_{i}-U_{N}\right|$$ (11)

Among them,  U is the voltage, $\;C_{\mu }$ is the expected deviation loss price of system voltage, which is 20 yuan.

#### 3.2.3. Constraints.

(1) Restrictions on the number of charging piles in the station:

$${n_{chmin}<n}_{ch}<n_{chmax}$$ (12)

Indicates the maximum value $n_{chmax}$ and the minimum value $n_{chmin}$ of the number of charging piles established in the charging station.

(2) Constraints on the number of charging stations

The number of charging stations is determined based on the demand for charging and the capacity of the charging stations. The prediction is based on the peak charging demand within the day and the capacity constraints of the charging stations. The calculation formula is as follows:


$$N_{CH}\geq N_{CH,min}$$ (13)

$$N_{CH,min}=\left|\frac{n_{ch}}{n_{ch,max}}\right|+\text{1}$$ (14)

(3) Power flow constraints

$$P_{Gh}-P_{ch}-V_{k}\sum_{j=\text{1}}^{N}V_{k}\left(G_{hk}\;\text{cos}{\;\theta }_{hk}+B_{hk}\;\text{sin}{\;\theta }_{hk}\right)=\text{0}$$ (15)

$$Q_{Gh}-Q_{ch}-V_{k}\sum_{j=\text{1}}^{N}V_{k}\left(G_{hk}\;\text{cos}{\;\theta }_{hk}+B_{hk}\;\text{sin}{\;\theta }_{hk}\right)=\text{0}$$ (16)

Where ∀hk∈L,∀h∈[1,N], $\;B_{hk}$ is the susceptance of the branch, $\;P_{Gh}$ and $P_{ch}$ represent the active power of the power generation and charging station load of the grid node, $\;Q_{Gh}$ and $Q_{ch}$ represent the reactive power of the power generation and charging station load of the grid node  h, respectively.

(4) Branch apparent power constraint


$$S_{hk}=\sqrt{P_{hk}^{\text{2}}+Q_{hk}^{\text{2}}}$$ (17)

$${|S}_{hk}|\leq {\text{S}}_{hk}^{max}$$ (18)

where ${\text{S}}_{hk}^{max}$ is the maximum apparent power in branch  h,k, $\;P_{hk}$ is the active power in branch  h,k, $\;Q_{hk}$ is the reactive power in branch  h,k.

(5) Node Voltage Constraints

$${\text{V}}_{h}^{min}\leq V_{h}\leq {\text{V}}_{h}^{max},\;\;\forall h\in [\text{1},N]$$ (19)

${\text{V}}_{h}^{min}$ is the lower limit of node voltage, $\;{\text{V}}_{h}^{max}$ is the upper limit of node voltage.

## 4. Chaotic non-uniform mutation-enhanced Artificial Hummingbird Algorithm (CAHA)

In electric-vehicle charging-station siting, the inclusion of multiple stakeholders and objectives continually increases problem complexity. Genetic algorithms, particle swarm optimization, and simulated annealing are frequently employed, yet their inherent structures often drive the search into local optima, compromising solution accuracy. To enhance convergence and global-search capability, this paper augments the Artificial Hummingbird Algorithm (AHA) with chaotic non-uniform mutation, proposing a novel Chaotic Non-uniform Mutation Artificial Hummingbird Algorithm (CAHA) specifically tailored for the multi-dimensional and multi-objective siting problem.

### 4.1. Tent Chaos map

Since the artificial hummingbird algorithm usually uses randomly generated data as the initial population position when solving optimization problems, it will have an adverse effect on the diversity of the population. Therefore, this paper introduces the Tent mapping [34]. The traversal of the Tent mapping shows uniform and random characteristics, which helps the algorithm to avoid falling into local optimal solutions more easily, thereby ensuring the diversity of the population and enhancing its global search capabilities. Therefore, this paper plans to use the Tent mapping to initialize the population and take, which is expressed as:



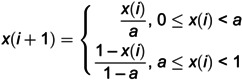

(20)


In addition to uniformity and randomness, the Tent chaotic map exhibits the following characteristics:

(1) Chaoticity: The Tent map displays chaotic behavior, whereby an infinitesimal perturbation in the initial condition can lead to an entirely different system trajectory.
(2) Piecewise Linearity: The Tent map is a piecewise function characterized by two distinct linear segments on the intervals [0, a] and [a, 1]. On [0, a] it behaves as a linear function with slope < 1, whereas on [a, 1] it is the mirror image of this linear function, again with slope < 1.

### 4.2. Crossover operator

The crossover operator usually refers to an operation used to generate new individuals in genetic algorithms and evolutionary computing. In these algorithms, individuals are often represented by chromosomes, and the crossover operator is a method of operating on these chromosomes.

The design of the crossover operator has a great influence on the convergence and search ability of the algorithm. This paper performs a crossover operation on the first 1/3 of the population with the best fitness and recalculates the fitness. When the fitness of the new individual is better than that of the original individual, it replaces the original individual, so that it has a certain probability of jumping out of the local optimal solution.

### 4.3. Non-uniform mutation operator

The non-uniform mutation operator is a nonlinear mutation operation whose mutation rate gradually decreases with the number of iterations, thereby introducing greater disturbances in early iterations and becoming more stable in later iterations. This approach helps to find a balance between global search and local search, thereby enhancing the practicality and convergence of the algorithm. In this article, we use a non-uniform mutation operator to perturb the position of the population so that individuals can search in a larger range at the beginning of the solution and perform fine search in local areas at the later stage. The non-uniform operator can be expressed as follows:



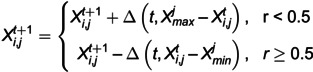

(21)


Where $X_{i,j}$ represents the element of the *i* th dimension of the *j* th individual. t is the current iteration number. $X_{max}$ and $X_{min}$ represent the maximum and minimum values of each dimension of the search space respectively. Δ(t,f) can be expressed as follows:


$$\Delta \left(t,f\right)=f\cdot \left(1-r^{{\left(1-\frac{t}{T}\right)}^{b}}\right)$$ (22)

Among them, T is the maximum number of iterations, b is the parameter that determines the non-uniformity.

### 4.4. Steps of CAHA

Based on the above improvements, this paper proposes a chaotic non-uniform mutation artificial hummingbird algorithm. In the population initialization process, we use Tent chaotic mapping to improve the diversity of the population; and for the artificial hummingbird algorithm [35], in order to solve the problem of easily falling into the local optimal solution, we introduce the crossover operator and non-uniform mutation operator, thereby improving the optimization search ability and convergence performance of the algorithm. The specific steps of the improved artificial hummingbird algorithm are as follows:

Step1: First, the positions (PopPos) and fitness (PopFit) of a group of hummingbirds are initialized by random generation. These positions are limited to a given search space range (between Low and Up).

Step2: Tent chaos optimization is used to obtain the initial population position. Based on the initial position, we calculate the ideal position and optimal fitness value of the population, the environmental safety signal, and initialize the access table;

Step3: In each iteration, the hummingbirds will fly and search according to certain rules. These rules include random selection of directions (diagonal, omnidirectional, axial flight), guided foraging and regional foraging strategies.

Step4: Periodically perform migration and foraging operations to maintain the diversity of the population. Within a specific number of iterations, select the hummingbird with the highest fitness to migrate and re-initialize its position randomly.

Step5: After a certain number of iterations, perform fitness sorting and perform crossover operations based on a part of the bees with the best fitness.

Step6: Introduce non-uniform mutation operators to perform non-uniform mutation operations on the positions of hummingbirds to increase the diversity of the search.

Step7: In each iteration, update the global optimal fitness (BestF) and the corresponding position (BestX).

Step8: Record the optimal fitness of each iteration for subsequent analysis of the algorithm’s performance.

### 4.5. Algorithm performance evaluation

In order to prove the supeanriority of the performance of the improved artificial hummingbird algorithm (CAHA), this section uses the improved artificial hummingbird algorithm (CAHA), artificial hummingbird algorithm (AHA) and particle swarm algorithm (PSO) to conduct simulation experiments on multiple test functions. The selected test functions include Sphere function, Ackley function, Griewank function and Rastrigin function.

**Fig 2:**
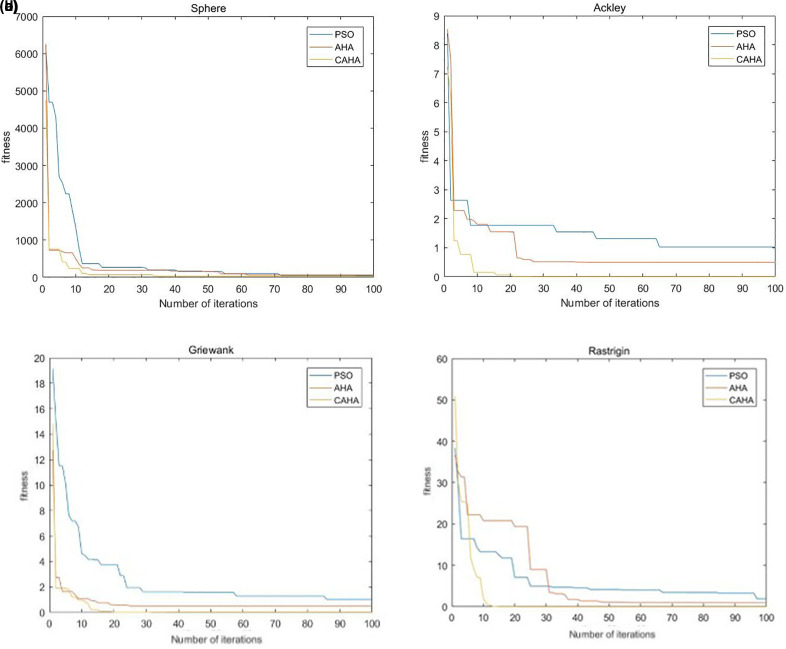
(a) Sphere function convergence curve. (b) Ackley function convergence curve. (c) Griewank function convergence curve. (d) Rastrigin function convergence curve.

For the improved CAHA algorithm, its core parameters include: selecting a population size of 30 and a maximum number of iterations of 100. The core parameters selected by AHA are consistent with the optimized AHA algorithm. In the design of the PSO algorithm, we selected 30 population particles, 5 particle dimensions, a maximum number of iterations of 100, and an inertia weight of 0.8. In order to intuitively compare the convergence of different algorithms and their ability to select the best solution, we show the convergence trends of the three algorithms on four test functions, as shown in Figs 2 (a), (b), (c) and (d).

The results indicate that CAHA converges rapidly on the multimodal Griewank and Rastrigin functions, while achieving higher prediction accuracy and stronger robustness on the unimodal Rosenbrock and Sphere functions without falling into local optima. The AHA enhanced by non-uniform mutation outperforms both the particle swarm algorithm and the conventional AHA in terms of iteration count and the ability to select the global optimum, successfully avoiding local optima. By initializing the population with a Tent chaotic map and incorporating crossover and non-uniform mutation operators, the proposed algorithm preserves the advantages of the original AHA while significantly improving overall performance.

### 4.6. Algorithm complexity

In the CAHA, the time complexity is derived as follows:

Tent-chaos initialization: O(N·D) for a one-off generation and mapping, where N is the population size and D the problem dimension. Hummingbird position update: O(N·D·T) over T iterations, with N individuals and D dimensions processed each generation. Crossover operator: O((N/3)·D) per generation, applied only to the top one-third of individuals. Non-uniform mutation: O(N·D) per generation, performing a per-dimension non-linear perturbation.

Hence, the overall time complexity of CAHA is O(N·D·T), the same asymptotic order as AHA and PSO. Empirically, for D ≤ 100 the running time of CAHA does not differ significantly from that of AHA or PSO.

## 5. Case analysis

A case study is conducted in a designated planning zone of Xi’an. The zone covers 72 km² and has a population density of 1200 inhabitants per square kilometre. Private-car ownership is 20% of the population, and 25% of these vehicles are electric. The area encompasses 36 residential-centroid demand points and 32 pre-selected candidate station sites. The candidate sites are assumed to correspond one-to-one with the IEEE 33-node test feeder. Coordinates of the demand points together with their associated EV counts are given in Table 2, while the precise coordinates of the candidate sites aligned with the IEEE 33-node system are listed in Table 3.

**Table 2:** Coordinate of demand point and number of electric vehicles.

Demand point number	Node horizontal coordinate (km)	Node ordinate (km)	Number of electric vehicles (Vehicle)
1	17.56	2.16	96
2	14.69	3.31	122
3	12.57	0.27	145
4	2.62	3.21	65
5	12.80	2.48	96
6	9.48	2.12	106
7	16.56	0.21	149
8	3.93	3.64	147
9	10.29	1.92	174
10	2.93	1.14	171
11	7.00	0.27	111
12	4.21	3.18	91
13	13.63	0.17	101
14	2.11	3.12	67
15	7.15	1.79	69
16	7.87	2.25	172
17	17.78	3.93	133
18	9.04	3.19	112
19	17.59	2.60	84
20	6.34	3.45	178
21	3.96	0.73	136
22	17.57	0.22	110
23	12.85	1.29	87
24	13.52	0.72	94
25	17.83	2.50	170
26	0.40	1.99	135
27	3.56	2.87	66
28	17.05	0.23	118
29	16.38	0.48	170
30	2.93	2.11	159
31	8.13	3.51	74
32	10.28	2.15	61
33	8.60	1.90	116
34	1.95	0.01	143
35	10.21	2.79	127
36	14.01	2.44	91

**Table 3:** Coordinate of demand point and number of electric vehicles.

Candidate point number	Node horizontal coordinate (km)	Node ordinate (km)	Corresponding Node
1	1.41	2.48	2
2	2.92	1.94	3
3	3.75	2.01	4
4	4.10	1.79	5
5	5.07	1.76	6
6	6.63	1.33	7
7	7.16	2.61	8
8	8.88	2.81	9
9	9.43	2.57	10
10	10.90	1.48	11
11	11.98	2.64	12
12	12.36	1.03	13
13	13.68	2.14	14
14	14.61	1.92	15
15	15.09	1.42	16
16	16.72	2.03	17
17	17.78	1.29	18
18	1.20	0.82	19
19	1.70	0.39	20
20	2.64	0.01	21
21	3.15	0.18	22
22	3.31	3.75	23
23	2.41	3.67	24
24	2.84	3.25	25
25	6.25	3.90	26
26	11.97	3.28	27
27	8.81	3.44	28
28	6.80	3.10	29
29	10.97	3.87	30
30	10.25	3.89	31
31	9.98	3.02	32
32	5.16	3.52	33

**Fig 3:**
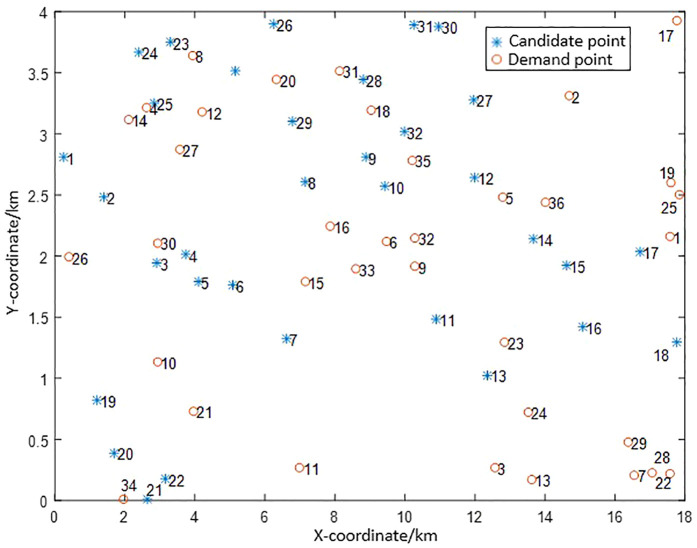
Topological map of planning area.

The corresponding topology of the planning zone is illustrated in Fig 3.

**Fig 4:**
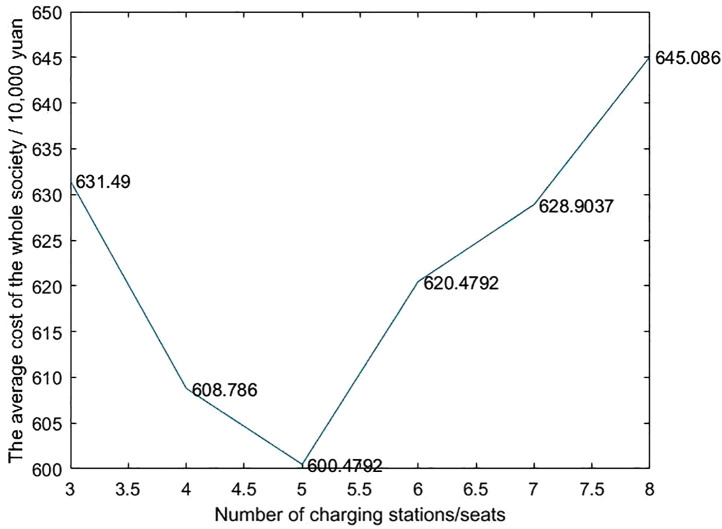
Comprehensive cost of different number of stations.

Through analysis, it is concluded that the number of charging stations is crucial to the development of the electric vehicle industry. Too few charging stations will reduce user experience and charging efficiency, which is not conducive to industrial development; too many charging stations will increase investment costs and waste land resources. Taking into account user experience, investment costs and social burden, as shown in Fig 4, building 5 charging stations can minimize the total social cost while meeting user needs. Specifically, 5 charging stations can optimize resource allocation, improve user experience, and promote the healthy development of the electric vehicle industry. It is an economically feasible solution. It is recommended to implement this plan in this planning area.

**Fig 5:**
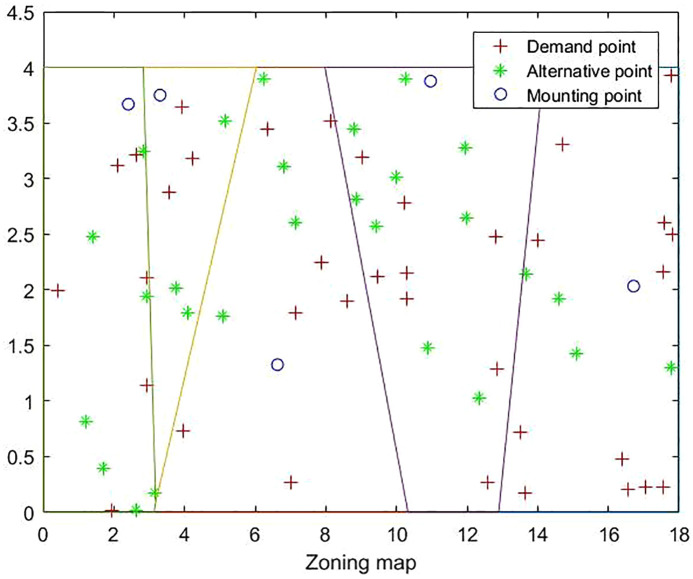
Optimal location scheme division.

The best site selection and capacity determination plan with the best overall total cost is shown in Fig 5, where the circular ring is the candidate site selection point. The number of charging piles in the selected site selection point and the ratio of charging piles under different charging modes are shown in Table 4.

**Table 4:** Location and capacity determination scheme and its ratio of fast and slow filling.

Charging station number	Select the candidate station number (site selection)	Number of charging piles (fixed capacity)	Charging speed ratio
1	17	14	0.7036
2	7	11	0.4850
3	23	7	0.1146
4	30	11	0.6649
5	24	10	0.3654

Through the data in Fig 5 and Table 4, we can clearly see that the planned area is divided into 5 service areas by 5 charging stations. Specifically, candidate station No. 17 is equipped with 14 charging piles, and its charging speed ratio is 0.7036; candidate station No. 7 is equipped with 11 charging piles, and its charging speed ratio reaches 0.4850; candidate station No. 23 is equipped with 7 charging piles, and its charging speed and speed ratio are both 0.1146; candidate station No. 30 is equipped with 11 charging piles, and its charging speed ratio reaches 0.6649; candidate station No. 24 is equipped with 10 charging piles, and its charging speed ratio reaches 0.3654.

To evaluate the robustness of the proposed algorithm, the number and proportion of fast/slow chargers at each candidate site were repeatedly varied; the CAHA was executed ten independent times. The resulting statistics are summarized in Table 5.

**Table 5:** CAHA test analysis.

Charging station number	Select the candidate station number (site selection)	Number of charging piles (fixed capacity)	Variance of the Number of Charging Piles	Charging speed ratio	Variance of the Fast-to-Slow Charger Ratio
1	17	14.0	0.40	0.704	8.3 × 10 ⁻ ⁶
2	7	11.1	0.29	0.486	6.4 × 10 ⁻ ⁶
3	23	7.0	0.22	0.115	4.9 × 10 ⁻ ⁶
4	30	11.1	0.29	0.666	5.6 × 10 ⁻ ⁶
5	24	10.0	0.22	0.365	4.2 × 10 ⁻ ⁶

As shown in Table 5, CAHA demonstrates strong robustness in charging-station siting. Regarding capacity allocation, the variance of the number of charging piles ranges from 0.22 to 0.4, indicating only minor fluctuations that remain within engineering tolerances. In the fast-to-slow charger ratio analysis, the relative deviation from the mean is less than 0.5%, underscoring the algorithm’s high stability.

To validate the practicality of the CAHA, comparative experiments were conducted among CAHA, the original Artificial Hummingbird Algorithm (AHA), Particle Swarm Optimization (PSO), and the widely-used multi-objective optimizer NSGA-II; the results are reported in Tables 6 and 7. As shown in Table 7, the solution obtained by the improved AHA (CAHA) yields a total cost of 1619.9631 across the four cost components, which is the lowest among all four algorithms. By contrast, the original AHA attains the smallest network-loss and voltage-deviation costs, yet its annual user travel-loss cost is more than twice that of the CAHA solution. PSO produces the highest value for every single cost component, resulting in the largest total cost.

**Table 6:** Four algorithms are used to obtain a fixed volume scheme.

Algorithm	Number of chargers for each type (fixed capacity)	Charging speed ratio
CAHA	14,11,7,11,10	0.7036,0.4850,0.1146,0.6649,0.3654
AHA	11,7,10,7,15	0.6802,0.0534,0.3567,0.4983,0.4344
PSO	13,14,6,11,8	0.5947,0.5657,0.7165,0.5113,0.7764
NSGA-II	12, 10, 8, 12, 9	0.6524, 0.4217, 0.1981,0.6035,0.3903

**Table 7:** Four algorithms are used to find the location scheme and various costs.

Algorithm	Site selection number (site selection)	Construction and operation cost/10,000 yuan	Annual user loss cost during charging/10,000 yuan	Network loss cost/10,000 yuan	Voltage offset cost/10,000 yuan	Total cost/10,000 yuan
CAHA	17,7,23,30,24	701.1417	181.0356	165.1915	572.5943	1619.9631
AHA	4,19,10,26,11	666.5718	388.1994	146.8078	525.4290	1727.008
PSO	15,22,27,12,13	708.4727	303.8732	178.1240	611.5207	1801.9906
NSGA-II	3, 16, 22, 29, 31	689.2318	245.7603	159.8457	548.7021	1 643.5337

In the context of EV-charging-station siting, the proposed multi-dimensional, multi-objective model demonstrates marked superiority over single-objective formulations. By simultaneously optimizing construction, maintenance, user travel, and system-loss costs, it furnishes a holistic solution that reconciles the often-conflicting requirements and constraints of diverse stakeholders—government agencies, EV users, and charging-station operators—allowing decision-makers to select the most appropriate compromise solution. Comparative results verifying these advantages are presented in Table 8.

**Table 8:** Cost comparison of single objective location and multi-dimensional multi-objective location.

Objective Function	Charging-Station Construction Cost	User Travel-Time Cost	Grid Loss Cost	Voltage Deviation Cost	Total Cost
Considering four objective functions	701.1417	181.0356	165.1915	572.5943	1617.9631
Optimizing only Objective Func 1	592.1720	512.1746	158.0091	565.1457	1827.5014
Optimizing only Objective Func 2	726.6714	133.9384	196.3154	651.3185	1708.2437
Optimizing only Objective Func 3 and 4	1427.9504	6797.5066	80.8082	308.6457	8614.9109

From Table 8, it can be seen that when only optimizing the cost objective function of charging station construction, this cost is the minimum value in the comparative experiment. However, in order to pursue the minimum cost of charging station construction, the user’s time consumption cost is ignored, making this cost 2.83 times higher than that of multi-objective site selection, which makes it extremely inconvenient for users to travel; However, when only considering the objective function of user travel cost, the construction cost and total cost of charging stations increase significantly compared to multi-dimensional and multi-objective site selection; If only considering the grid losses and voltage offsets of the power grid, the construction cost of charging stations will be 2.04 times that of multi-dimensional multi-objective site selection, and the user’s time consumption cost will even skyrocket to 37.55 times that of multi- dimensional multi-objective site selection. Even more unreasonable is the situation where five charging stations are concentrated in one place to extremely control grid losses and voltage offsets during site selection. The multi-dimensional and multi-objective site selection in this article not only minimizes the total cost, but also balances the four objective functions, keeping them within a more reasonable cost range.

To verify the generalization and robustness of the algorithm, the CAHA algorithm and the multi-dimensional multi-objective location game process are extended to more complex power grids. In this paper, a larger-scale area is selected for planning, with 72 demand points and 58 candidate points based on the planning. The coordinate data of each point and the number of electric vehicles are shown in Attachment 1. The IEEE 84 system is selected as the test system. After economic analysis, the final optimal location result is 21 candidate locations, as shown in Table 9.

**Table 9:** Location and capacity determination scheme and its ratio of fast and slow filling.

Charging station number	Select the candidate station number (site selection)	Number of charging piles (fixed capacity)	Charging speed ratio
1	5	15	0.65
2	12	10	0.45
3	18	8	0.30
4	23	7	0.25
5	30	11	0.60
6	35	9	0.50
7	40	10	0.40
8	45	12	0.65
9	50	13	0.70
10	55	14	0.75
11	5	15	0.65
11	58	15	0.80
12	10	16	0.85
13	15	17	0.90
14	20	18	0.95
15	25	19	1.00
16	30	20	1.05
17	35	21	1.10
18	40	22	1.15
19	45	23	1.20
20	50	24	1.25
21	55	25	1.30

To test the robustness of the algorithm, the number and proportion of charging piles at each candidate site were repeatedly varied; the CAHA method was executed ten independent times. Table 10 summarizes the resulting statistics.

**Table 10:** CAHA test analysis.

Charging station number	Select the candidate station number (site selection)	Number of charging piles (fixed capacity)	Variance of the Number of Charging Piles	Charging speed ratio	Variance of the Fast-to-Slow Charger Ratio
1	5	15.0	0.30	0.65	7.0 × 10 ⁻ ⁶
2	12	10.1	0.25	0.45	6.0 × 10 ⁻ ⁶
3	18	8.0	0.20	0.30	5.0 × 10 ⁻ ⁶
4	23	7.0	0.15	0.25	4.0 × 10 ⁻ ⁶
5	30	11.0	0.22	0.60	6.5 × 10 ⁻ ⁶
6	35	9.0	0.18	0.50	5.5 × 10 ⁻ ⁶
7	40	10.0	0.20	0.40	5.0 × 10 ⁻ ⁶
8	45	12.0	0.25	0.65	6.0 × 10 ⁻ ⁶
9	50	13.0	0.28	0.70	6.8 × 10 ⁻ ⁶
10	55	14.0	0.30	0.75	7.2 × 10 ⁻ ⁶
11	58	15.0	0.32	0.80	7.5 × 10 ⁻ ⁶
12	10	16.0	0.35	0.85	7.8 × 10 ⁻ ⁶
13	15	17.0	0.38	0.90	8.0 × 10 ⁻ ⁶
14	20	18.0	0.40	0.95	8.2 × 10 ⁻ ⁶
15	25	19.0	0.42	1.00	8.5 × 10 ⁻ ⁶
16	30	20.0	0.45	1.05	8.8 × 10 ⁻ ⁶
17	35	21.0	0.48	1.10	9.0 × 10 ⁻ ⁶
18	40	22.0	0.50	1.15	9.2 × 10 ⁻ ⁶
19	45	23.0	0.52	1.20	9.4 × 10 ⁻ ⁶
20	50	24.0	0.55	1.25	9.6 × 10 ⁻ ⁶
21	55	25.0	0.58	1.30	9.8 × 10 ⁻ ⁶

As shown in Table 10, CAHA demonstrates strong robustness in charging-station siting. Regarding capacity allocation, the variance of the number of charging piles ranges from 0.15 to 0.58, indicating only minor fluctuations that remain within engineering tolerances. In the fast-to-slow charger ratio analysis, the relative deviation from the mean is less than 0.5%, underscoring the algorithm’s high stability.

To validate the practicality of the CAHA, comparative experiments were conducted among CAHA, the original Artificial Hummingbird Algorithm (AHA), Particle Swarm Optimization (PSO), and the widely-used multi-objective optimizer NSGA-II; the results are reported in Tables 11 and 12. As shown in Table 12, the solution obtained by the improved AHA (CAHA) yields a total cost of 6432.45 across the four cost components, which is the lowest among all four algorithms. By contrast, the original AHA attains the smallest network-loss and voltage-deviation costs, yet its annual user travel-loss cost is more than twice that of the CAHA solution. NSGA-II produces the highest value for every single cost component, resulting in the largest total cost.

**Table 11:** Four algorithms are used to obtain a fixed volume scheme.

Algorithm	Number of chargers for each type (fixed capacity)	Charging speed ratio
CAHA	15, 10, 8, 7, 11, 9, 10, 12, 13, 14, 15, 16, 17, 18, 19, 20, 21, 22, 23, 24, 25	0.65, 0.45, 0.30, 0.25, 0.60, 0.50, 0.40, 0.65, 0.70, 0.75, 0.80, 0.85, 0.90, 0.95, 1.00, 1.05, 1.10, 1.15, 1.20, 1.25, 1.30
AHA	14, 9, 7, 6, 10, 8, 9, 11, 12, 13, 14, 15, 16, 17, 18, 19, 20, 21, 22, 23, 24	0.60, 0.40, 0.25, 0.20, 0.55, 0.45, 0.35, 0.60, 0.65, 0.70, 0.75, 0.80, 0.85, 0.90, 0.95, 1.00, 1.05, 1.10, 1.15, 1.20, 1.25
PSO	16, 11, 9, 8, 12, 10, 11, 13, 14, 15, 16, 17, 18, 19, 20, 21, 22, 23, 24, 25, 26	0.68, 0.48, 0.35, 0.30, 0.65, 0.55, 0.45, 0.70, 0.75, 0.80, 0.85, 0.90, 0.95, 1.00, 1.05, 1.10, 1.15, 1.20, 1.25, 1.30, 1.35
NSGA-II	15, 10, 8, 7, 11, 9, 10, 12, 13, 14, 15, 16, 17, 18, 19, 20, 21, 22, 23, 24, 25	0.63, 0.43, 0.30, 0.25, 0.58, 0.48, 0.38, 0.63, 0.68, 0.73, 0.78, 0.83, 0.88, 0.93, 0.98, 1.03, 1.08, 1.13, 1.18, 1.23, 1.28

**Table 12:** Four algorithms are used to find the location scheme and various costs.

Algorithm	Site selection number (site selection)	Construction and operation cost/10,000 yuan	Annual user loss cost during charging/10,000 yuan	Network loss cost/10,000 yuan	Voltage offset cost/10,000 yuan	Total cost/10,000 yuan
CAHA	5, 12, 18, 23, 30, 35, 40, 45, 50, 55, 58, 10, 15, 20, 25, 30, 35, 40, 45, 50, 55	2961.85	741.15	611.10	2118.15	6432.25
AHA	4, 11, 17, 22, 29, 34, 39, 44, 49, 54, 57, 9, 14, 19, 24, 29, 34, 39, 44, 49, 54	2591.20	1894.40	669.45	2214.45	9369.50
PSO	6, 13, 19, 24, 31, 36, 41, 46, 51, 56, 59, 11, 16, 21, 26, 31, 36, 41, 46, 51, 56	2788.20	570.20	739.45	2394.45	6492.30
NSGA-II	5, 12, 18, 23, 30, 35, 40, 45, 50, 55, 58, 10, 15, 20, 25, 30, 35, 40, 45, 50, 55	5281.80	25050.25	375.45	1140.45	32048.00

The results of the multi-dimensional multi-objective location cost strategy tested in a complex system are shown in Table 13.

**Table 13:** Cost comparison of single objective location and multi-dimensional multi-objective location.

Objective Function	Charging-Station Construction Cost	User Travel-Time Cost	Grid Loss Cost	Voltage Deviation Cost	Total Cost
Considering four objective functions	2961.85	741.15	611.10	2118.15	6432.25
Optimizing only Objective Func 1	2591.20	1894.40	669.45	2214.45	9369.50
Optimizing only Objective Func 2	2788.20	570.20	739.45	2394.45	6492.30
Optimizing only Objective Func 3 and 4	5281.80	25050.25	375.45	1140.45	32048.00

As can be seen from the above tables, the costs based on multi-factor and multi-objective location optimization results are superior to decision-making results that only consider single objective functions. Therefore, under both complex and non-complex multi-condition tests, the CAHA model and the multi-dimensional multi-objective location game model can achieve good results and have strong generalization and robustness.

The current validation of the proposed CAHA method are confined to the IEEE 33 and IEEE 84-node feeder, which are relatively small and highly aggregated. While the Xi’an 72 km² planning case offers a real-world setting, it still represents only a medium-scale distribution network. To strengthen the impact and generalizability of the findings, future work will (1) test CAHA on larger, real-world datasets—specifically 1000-plus-node urban grids and extensive highway networks—and (2) incorporate high-uncertainty factors such as time-varying loads, renewable-generation fluctuations, and dynamic electricity prices to assess robustness and scalability under more complex operating conditions.

## Conclusion

This study proposes an electric vehicle charging station location and capacity optimization model based on the Improved Artificial Hummingbird Algorithm (CAHA), aiming to solve problems such as charging station construction cost, user charging time loss, grid loss, and voltage deviation through multi-objective optimization. Through calculation and verification, the following conclusions can be drawn as follows:

(1) The four-objective function for the three-party game between suppliers, users and the grid is established, namely the construction cost of the charging station, the annual user loss cost during the charging journey, the network loss cost and the voltage offset cost. By introducing the ratio of fast and slow charging piles, the design of the charging station is more reasonable while the cost is controlled, which is in line with the future trend.
(2) The proposed CAHA algorithm is based on the AHA algorithm and integrates Tent’s chaos theory, crossover operator and non-uniform mutation operator, which significantly improves the convergence speed of the algorithm and its ability to escape from the local optimal solution. Compared with the AHA algorithm, CAHA has demonstrated higher execution efficiency and better optimization search capabilities in the planning process of electric vehicle charging stations.
(3) With the rapid development of electric vehicle infrastructure, it has greatly improved the actual charging station site selection project and saved costs.

Although the model has shown good optimization performance and robustness in theory and experiments, there are some limitations in practical applications, such as simplifying assumptions, static demand assumptions, simplifying grid constraints, and issues with computational cost and scalability. Future research directions include dynamic demand modeling, renewable energy integration, multi-objective optimization algorithm improvement, practical case verification, and policy and market factor analysis to enhance the practicality and foresight of research and provide stronger support for the planning and construction of electric vehicle charging infrastructure.

## Supporting information

S1 File IEEE84 DATA. (DOCX)
