# Supplementary material for: Improved artificial hummingbird algorithm for electric vehicle charging station fast and slow charging location determination method
Source: PLoS One. 2025 Sep 30;20(9):e0332872. doi: 10.1371/journal.pone.0332872 (PMC12483277; doi:10.1371/journal.pone.0332872)
Supplement: S1 File — (DOCX) [file pone.0332872.s001.docx]

Using the IEEE 84 system for estimation, the planning demand points and the corresponding data of the demand points are as follows.

Table1 Coordinate of demand point and number of electric vehicles (IEEE 84)

| Demand point number | Node horizontal coordinate (km) | Node ordinate  (km) | Number of electric vehicles (Vehicle) |
| --- | --- | --- | --- |
| 1 | 1.41 | 2.48 | 102 |
| 2 | 2.92 | 1.94 | 116 |
| 3 | 3.75 | 2.01 | 129 |
| 4 | 4.10 | 1.79 | 142 |
| 5 | 5.07 | 1.76 | 155 |
| 6 | 6.63 | 1.33 | 169 |
| 7 | 7.16 | 2.61 | 182 |
| 8 | 8.88 | 2.81 | 179 |
| 9 | 9.43 | 2.57 | 166 |
| 10 | 10.90 | 1.48 | 154 |
| 11 | 11.98 | 2.64 | 141 |
| 12 | 12.36 | 1.03 | 128 |
| 13 | 13.68 | 2.14 | 116 |
| 14 | 14.61 | 1.92 | 103 |
| 15 | 15.09 | 1.42 | 90 |
| 16 | 16.72 | 2.03 | 78 |
| 17 | 17.78 | 1.29 | 65 |
| 18 | 1.20 | 0.82 | 52 |
| 19 | 1.70 | 0.39 | 39 |
| 20 | 2.64 | 0.01 | 27 |
| 21 | 3.15 | 0.18 | 14 |
| 22 | 3.31 | 3.75 | 107 |
| 23 | 2.41 | 3.67 | 125 |
| 24 | 2.84 | 3.25 | 142 |
| 25 | 6.25 | 3.90 | 159 |
| 26 | 11.97 | 3.28 | 176 |
| 27 | 8.81 | 3.44 | 193 |
| 28 | 6.80 | 3.10 | 181 |
| 29 | 10.97 | 3.87 | 168 |
| 30 | 10.25 | 3.89 | 156 |
| 31 | 9.98 | 3.02 | 143 |
| 32 | 5.16 | 3.52 | 130 |
| 33 | 1.95 | 0.01 | 117 |
| 34 | 1.90 | 1.90 | 105 |
| 35 | 1.21 | 2.79 | 92 |
| 36 | 1.40 | 2.44 | 79 |
| 37 | 1.50 | 2.50 | 67 |
| 38 | 1.60 | 2.60 | 54 |
| 39 | 1.70 | 2.70 | 41 |
| 40 | 1.80 | 2.80 | 29 |
| 41 | 1.90 | 2.90 | 16 |
| 42 | 2.00 | 3.00 | 113 |
| 43 | 2.10 | 3.10 | 130 |
| 44 | 2.20 | 3.20 | 146 |
| 45 | 2.30 | 3.30 | 162 |
| 46 | 2.40 | 3.40 | 178 |
| 47 | 2.50 | 3.50 | 195 |
| 48 | 2.60 | 3.60 | 182 |
| 49 | 2.70 | 3.70 | 169 |
| 50 | 2.80 | 3.80 | 156 |
| 51 | 2.90 | 3.90 | 144 |
| 52 | 3.00 | 4.00 | 131 |
| 53 | 3.10 | 4.10 | 118 |
| 54 | 3.20 | 4.20 | 106 |
| 55 | 3.30 | 4.30 | 93 |
| 56 | 3.40 | 4.40 | 80 |
| 57 | 3.50 | 4.50 | 68 |
| 58 | 3.60 | 4.60 | 55 |
| 59 | 3.70 | 4.70 | 42 |
| 60 | 3.80 | 4.80 | 30 |
| 61 | 3.90 | 4.90 | 17 |
| 62 | 4.00 | 5.00 | 119 |
| 63 | 4.10 | 5.10 | 136 |
| 64 | 4.20 | 5.20 | 152 |
| 65 | 4.30 | 5.30 | 168 |
| 66 | 4.40 | 5.40 | 184 |
| 67 | 4.50 | 5.50 | 169 |
| 68 | 4.60 | 5.60 | 155 |
| 69 | 4.70 | 5.70 | 142 |
| 70 | 4.80 | 5.80 | 129 |
| 71 | 4.90 | 5.90 | 116 |
| 72 | 5.00 | 6.00 | 182 |

Table 2 Coordinate of demand point and number of electric vehicles (IEEE 84)

| Candidate point number | Node horizontal coordinate (km) | | | Node ordinate (km) | Corresponding Node | |
| --- | --- | --- | --- | --- | --- | --- |
| 1 | | 1.41 | 2.48 | | | 2 |
| 2 | | 2.92 | 1.94 | | | 3 |
| 3 | | 3.75 | 2.01 | | | 4 |
| 4 | | 4.10 | 1.79 | | | 5 |
| 5 | | 5.07 | 1.76 | | | 6 |
| 6 | | 6.63 | 1.33 | | | 7 |
| 7 | | 7.16 | 2.61 | | | 8 |
| 8 | | 8.88 | 2.81 | | | 9 |
| 9 | | 9.43 | 2.57 | | | 10 |
| 10 | | 10.90 | 1.48 | | | 11 |
| 11 | | 11.98 | 2.64 | | | 12 |
| 12 | | 12.36 | 1.03 | | | 13 |
| 13 | | 13.68 | 2.14 | | | 14 |
| 14 | | 14.61 | 1.92 | | | 15 |
| 15 | | 15.09 | 1.42 | | | 16 |
| 16 | | 16.72 | 2.03 | | | 17 |
| 17 | | 17.78 | 1.29 | | | 18 |
| 18 | | 1.20 | 0.82 | | | 19 |
| 19 | | 1.70 | 0.39 | | | 20 |
| 20 | | 2.64 | 0.01 | | | 21 |
| 21 | | 3.15 | 0.18 | | | 22 |
| 22 | | 3.31 | 3.75 | | | 23 |
| 23 | | 2.41 | 3.67 | | | 24 |
| 24 | | 2.84 | 3.25 | | | 25 |
| 25 | | 6.25 | 3.90 | | | 26 |
| 26 | | 11.97 | 3.28 | | | 27 |
| 27 | | 8.81 | 3.44 | | | 28 |
| 28 | | 6.80 | 3.10 | | | 29 |
| 29 | | 10.97 | 3.87 | | | 30 |
| 30 | | 10.25 | 3.89 | | | 31 |
| 31 | | 9.98 | 3.02 | | | 32 |
| 32 | | 5.16 | 3.52 | | | 33 |
| 33 | | 1.95 | 0.01 | | | 34 |
| 34 | | 1.90 | 1.90 | | | 35 |
| 35 | | 1.21 | 2.79 | | | 36 |
| 36 | | 1.40 | 2.44 | | | 37 |
| 37 | | 1.50 | 2.50 | | | 38 |
| 38 | | 1.60 | 2.60 | | | 39 |
| 39 | | 1.70 | 2.70 | | | 40 |
| 40 | | 1.80 | 2.80 | | | 41 |
| 41 | | 1.90 | 2.90 | | | 42 |
| 42 | | 2.00 | 3.00 | | | 43 |
| 43 | | 2.10 | 3.10 | | | 44 |
| 44 | | 2.20 | 3.20 | | | 45 |
| 45 | | 2.30 | 3.30 | | | 46 |
| 46 | | 2.40 | 3.40 | | | 47 |
| 47 | | 2.50 | 3.50 | | | 48 |
| 48 | | 2.60 | 3.60 | | | 49 |
| 49 | | 2.70 | 3.70 | | | 50 |
| 50 | | 2.80 | 3.80 | | | 51 |
| 51 | | 2.90 | 3.90 | | | 52 |
| 52 | | 3.00 | 4.00 | | | 53 |
| 53 | | 3.10 | 4.10 | | | 54 |
| 54 | | 3.20 | 4.20 | | | 55 |
| 55 | | 3.30 | 4.30 | | | 56 |
| 56 | | 3.40 | 4.40 | | | 57 |
| 57 | | 3.50 | 4.50 | | | 58 |
| 58 | | 3.60 | 4.60 | | | 59 |
